# Neuropathic Pain Management Using Novel Biologics in Moderate-to-Severe Hidradenitis Suppurativa

**DOI:** 10.1007/s11916-026-01551-y

**Published:** 2026-08-31

**Authors:** Josephine M. Feeney, Nikayla A. Henderson, Tanisha M. Patel, Anna K. Ardoin, Ahmed I. Anwar, Alaa Abd-Elsayed, Alan D. Kaye

**Affiliations:** 1 School of Medicine, Louisiana State University Health Sciences Center at Shreveport, 1501 Kings Highway, Shreveport, LA 71103 USA; 2 Department of Anesthesiology, University of Wisconsin School of Medicine and Public Health, 600 Highland Avenue, B6/319 CSC, Madison, WI 53792-3272 USA; 3 Department of Anesthesiology, Louisiana State University Health Sciences Center at Shreveport, 1501 Kings Highway, Shreveport, LA 71103 USA

**Keywords:** Hidradenitis suppurativa, IL-17A inhibitors, IL-17A/IL-17F inhibitors, IL-1α/β inhibitors, Neuropathic pain, TNF-α inhibitors, Biologics

## Abstract

**Purpose of Review:**

Hidradenitis suppurativa (HS) is an extremely painful skin condition with both nociceptive and neuropathic components. Biologics may offer superior neuropathic pain relief and impede worsening of this disease compared to other treatments.

**Recent Findings:**

Of the three currently FDA-approved biologics (adalimumab, bimekizumab, and secukinumab), bimekizumab shows the largest absolute reduction in skin pain and the earliest onset of pain improvement.

**Summary:**

This review explores the pathophysiology and causes of neuropathic pain development in HS, highlights current FDA-approved and emerging biologics for its management, and raises awareness of neuropathic pain in HS. Clinical trial data assessing pain are unstandardized, and further elucidation of pain outcomes using standardized assessments of neuropathic pain is crucial for determining whether biologics targeting mediators that drive neuropathic pain development are effective in treating this aspect of HS.

## Introduction

Hidradenitis suppurativa (HS) is a skin condition characterized by recurrent nodules that can be profoundly debilitating for many patients. These painful lesions primarily originate within hair follicles in intertriginous (flexural) areas, most commonly the axilla, submammary region, inner thighs, groin, and buttocks [1]. Although some nodules may resolve spontaneously, others progress to form abscesses, draining sinus tracts, and ultimately heal with considerable scarring [2]. While the exact cause of this chronic skin disorder is unknown, some cases are passed down through autosomal dominant inheritance, and others are sporadic [3]. The global prevalence of HS ranges from 0.053% to 4.1%. This range likely reflects regional differences as well as frequent misdiagnosis as bacterial abscesses and underdiagnosis [4]. However, some researchers posit that HS’s actual prevalence is closer to 1% [5]. In North America, women are more likely to be affected by HS than men, whereas a male predominance has been reported in some Asian countries, including Japan and South Korea [4]. Other populations disproportionately affected by HS include individuals with Down syndrome, who are at an approximately fivefold increased risk of facing HS [6].

Additionally, in the United States, Black individuals have a threefold increased risk of HS compared to White individuals. Disease onset most commonly occurs between 18 and 39 years of age [6]. In the US, HS-specific direct costs range from $258 to $8,078 per patient per year, reflecting the significant economic burden of this dermatological disease [7].

An HS diagnosis is made based on clinical features because there is no pathognomonic test. Ultrasound imaging and histopathological studies can further aid in identifying the extent of the disease [2]. However, underdiagnosis is common due to substantial phenotypic diversity. Diagnosis is frequently delayed by 7–10 years on average [2]. Disease severity in HS is classified using the Hurley staging system, which ranges from Stage I (isolated abscess formation without tunneling or scarring) to Stage III (diffuse abscess formation, tunneling, and scarring) [8]. Moderate-to-severe disease, corresponding to Hurley Stages II and III, is associated with a higher pain burden, making this population the focus of the present review. Management of HS depends on severity and disease progression. Early sinus tracts are managed surgically with deroofing, while severe scarring requires wide local excision [9]. Because biologics (pharmaceutical therapies derived from living organisms) target the underlying pathologic mechanisms driving HS, they have become integral to managing more advanced disease [10, 11]. As of July 2026, three biologics are Food and Drug Administration (FDA) approved specifically for Hurley stage II and III disease: adalimumab, bimekizumab, and secukinumab. A 2025 meta-analysis of 25 randomized clinical trials found that adalimumab has similar HiSCR50 values to both IL-17 inhibitors and emerging biologics [12]. Regardless, there remains a need for novel biologics because TNF-α inhibitors like adalimumab do not work for all patients. Furthermore, up to half of initial responders lose their response over time [13].

HS can substantially lower the quality of life because patients suffer from severe pain, inflammation, malodor, decreased mobility, and cosmetic deformities. Among dermatological diseases, HS has one of the worst effects on well-being and life satisfaction [6]. Patients with HS have been shown to have a higher risk for suicide, mental health disorders, substance use disorders, cardiovascular events, and overall mortality [2, 14]. Pain is the most burdensome HS symptom and correlates positively with greater disease severity [15]. In one study of 138 patients, pain was most often described as “shooting” (83%), “itchy” (79%), and “blinding” (75%), descriptors that suggest pain is more complex than simple tissue injury alone [16]. Pain in HS has both nociceptive and neuropathic components [17]. Nociceptive pain in HS is driven by inflammation. This is caused by the breakdown of blocked hair follicles, which release DAMPs and PAMPs that activate members of the innate immune system, triggering the release of pro-inflammatory cytokines that activate nociceptors in the periphery [18, 19]. In contrast, neuropathic pain, a type of pathological pain, is related to nervous system damage, potentially due to chronic inflammation that alters peripheral sensory nerves [20]. Neuropathic pain is classically associated with postherpetic neuralgia (PHN) and painful diabetic peripheral neuropathy (DPN) [21]. A growing body of evidence suggests neuropathic pain is also common in HS: one study found that 64% of HS patients across all Hurley stages exhibited objective signs of neuropathic pain, including allodynia and paradoxical thermal sensations [22].

Despite this, most HS clinical trials assess pain using the NRS (numeric rating scale), which does not distinguish between neuropathic and nociceptive pain [23]. This is a significant gap given that up to 64% of patients with HS report a neuropathic pain component that also leaves the neuropathic-pain-modulating potential of biologics unmeasured [22]. The purpose of the present investigation, therefore, is to evaluate the neuropathic pain burden in moderate-to-severe HS and to examine the role of current and emerging biologics in its management.

## Methods

This narrative review was conducted using a literature search of the PubMed and ScienceDirect databases to identify studies examining neuropathic pain in hidradenitis suppurativa, as well as new and emerging drug therapies, especially biologics. The literature search was conducted in July 2026 for English-language articles. Articles published up to July 2026 were considered. Initial search results were screened by title and abstract. Potentially relevant studies were selected for full-text review based on their relevance to the management of HS neuropathic pain with biologics. Papers that were not peer-reviewed publications were excluded. Articles were identified using relevant keywords, which included but were not limited to “hidradenitis suppurativa”, “neuropathic pain, “nociceptive pain”, “TNF-α inhibitors,” “IL-17A inhibitors,” “IL-17A/IL-17F inhibitors,” “IL-1α/β inhibitors,” “IL-36 receptor inhibitors,” “adalimumab,” “bimekizumab,” and “secukinumab.” Studies were selected based on their relevance to the pathophysiology of hidradenitis suppurativa, the burden of neuropathic pain, and clinical trials of biologics for HS treatment. For the primary analysis, phase II and phase III clinical trials of different biologics were included. Biologics were excluded if pain was not evaluated using any method or if HiSCR50 was not reported. High-quality secondary syntheses, which included meta-analyses and systematic reviews, were prioritized for background information.

## Biological Basis of Pain in Hidradenitis Suppurativa

### Pathophysiology of HS

The pathophysiology of HS involves a complex interplay among several processes, including hyperkeratosis of the follicles, dysregulated immune stimulation, acute and chronic inflammation, and bacterial infection [2, 14]. HS may result from occluded follicles, which lead to excess keratin building up in the infundibulum part of the hair follicle and an increased number of follicular epithelial cells. This process causes entrapment of cellular debris. Then, the hair follicles swell and rupture [4]. Although the precise mechanism underlying HS remains incompletely understood, activation of inflammatory and immune pathways is likely to be a key driver of the advancement of this condition. Melchor et al. identified multiple cytokines and biomarkers that are related to HS pathogenesis, including both established markers reported in previous studies and newly identified biomarkers [24]. Identified proinflammatory cytokines included IL-1, IL-17, IL-23, and TNF-α, along with the anti-inflammatory cytokine IL-10. Potential biomarkers identified included granulocyte colony-stimulating factor (G-CSF), chitinase-3-like protein 1 (YKL-40), nucleotide-binding oligomerization domain 2 (NOD2), and the complement system [24]. Although many of these cytokines and biomarkers are implicated in other autoimmune and chronic inflammatory disorders, they give important insights into the progression of HS and may serve as potential therapeutic targets.

### Pathophysiology of Nociceptive Pain in HS

Nociceptive pain in HS is characterized as a throbbing pain caused by tissue destruction that primarily ensues from pro-inflammatory mediators activated by ruptured hair follicles, which release DAMPs from the damaged tissue cells and PAMPs from bacteria. These danger signals activate innate immune cells to release the cytokines IL-17, IL-1β, and TNF-α and lower the activation threshold for nociceptors [1, 19, 25, 26]. Nociceptors respond to increased mechanical inputs via peripheral Aδ and C nerve fibers as pus accumulates within inflamed tissue spaces. Ongoing nociceptor activation occurs from tissue damage that leads to epithelialized tunnel formation, where chronic physical friction and chemical stimulation lead to inflammation and irritation even in the absence of a flare [19].

### Pathophysiology of Neuropathic Pain in HS

Neuropathic pain in HS is thought to arise from the cycles of nodules and scarring [27]. Patients often described their symptoms as sharp, shooting, hot, itchy, and taut [16]. At the forefront of these. On the molecular level, symptoms are dysregulated immune responses involving cytokines. TNF-α, IL-1β, IL-1α, and IL-17 are the most well-recognized cytokines believed to contribute to the progression of HS and neuropathic pain [24]. Specifically, TNF-α is a proinflammatory cytokine that can prompt the expression of inducible nitric oxide synthase and cyclooxygenase [28, 29]. This leads to an overproduction of nitric oxide and prostaglandins within HS lesions, sensitizing local nerve endings and causing hyperexcitability, which leads to the inflamed tissue developing into neuropathic pain [29, 30]. In HS, the tissue transcriptome shows heightened expression of genes involved in inflammation, neutrophil activation, and motility, as well as IL-17 A and IL-17 F [31]. Involved heavily in neutrophil recruitment, both IL-17 A and IL-17 F were found to be elevated in lesional skin compared to control skin [31]. Once cleaved by the inflammasome to its activated form, IL-1b contributes to the pathogenesis of HS by eliciting the increased production of matrix metalloproteinases and chemokines, contributing to the destructive environment [32]. While IL-1α acts through a similar receptor mechanism, it showed only a slight increase compared to healthy control skin [32].

Central sensitization in HS is associated with a lowered threshold for pain processing, which varies among patients [33]. Central sensitization is scored via the Central Sensitization Inventory, with the scale being from 0 to 100. For central sensitization to be present, patients must receive a score equal to or greater than 40 [33]. In a case-control study matching 100 HS patients by age and sex, HS patients had a higher average CSI score (31 vs. 25) and an odds ratio of 4.446 for a positive CSI [33, 34].

Preliminary research indicates that structural changes in the nerves are also occurring in patients with HS. In a recent abstract, researchers analyzed 6 out of 10 of their cohort, using 3 mm punch biopsies to analyze cutaneous tissues, and reported a 50 to 70% reduction in intraepidermal nerve fiber density in skin close to nodules and tunnels, in comparison to skin not adjacent to lesions in the patients [35].

Along with structural changes and sensitization, depression also alters pain perception in this condition. Depression is highly prevalent in this population. A cross-sectional study including 38,140 adults and 1,162 pediatric patients found that depression was present in 30% (95% CI, 29.6–30.5) of adults with HS, compared to 16.9% (95% CI, 16.7–17.1) in controls [36]. In pediatric patients, depression was prevalent in 11.7% (95% CI, 10.0–13.7), versus 4.1% (95% CI, 2.51–4.02) in controls [36]. In describing pain, patients with HS and the comorbid conditions depression or anxiety, used terms with higher values, 65% versus 57% in controls (*P* = 0.015), and 65% compared to 57% (*P* = 0.004) respectively [16]. These findings highlight not only the physical burden experienced by these patients, but also the mental burden.

Clinically, neuropathic pain contributes significantly to the quality-of-life impairment seen in HS. The pain can persist beyond active lesions, affecting sleep, daily movement, and mood, making its recognition vital for comprehensive patient care [37].

## Suggestions for Clinicians: Clinical Assessment of Neuropathic Pain in HS

Assessment of neuropathic pain in HS often employs symptom checklists and physical testing to screen for specific features. One example of such a checklist is the PainDETECT survey. The PainDETECT survey asks how often patients experience sensory symptoms and about the course of the pain. It uses a categorical scale that leaves a “grey area” in which neuropathic pain is possible but not confirmed, yet it distinguishes neuropathic from non-neuropathic pain [38].utilizes a categorical scale that leaves a “grey area” in which neuropathic pain is possible but not confirmed, but it does distinguish neuropathic from non-neuropathic pain [38]. Because this survey has only 9 items and is self-reported by patients, it is likely the most feasible instrument for detecting neuropathic pain in patients with HS. The PainDETECT survey is free and takes 5 min to complete [38, 39].

Quantitative Sensory Testing (QST) comprises various tests that employ cotton wisps, pins, tuning forks, von Frey hairs, and pressure devices to assess thermal and mechanical detection, as well as allodynia [40]. A recent QST study shows that patients with chronic HS experience large sensory deficits to cold and warmth, while there was mechanical hypersensitivity in some who have hyperalgesia [22]. Researchers propose that nociceptive pain begins with inflammation and that neuropathic pain develops from repeated inflammation that results in structural changes in the nerves [41]. However, QST takes 30 min to over an hour to complete, requires specialized equipment, and is available only in specialized centers, which makes it impractical for evaluating neuropathic pain in HS in a clinic [42].

Other forms of questionnaires measure intensity by assessing patients’ interpretation of their pain. The Dermatology Life Quality Index (DLQI) was a 10-question survey developed in 1994 and assesses how skin conditions impact the everyday life of affected patients [43]. One caveat to this is that only one question is asked to assess pain, in which the pain descriptors include terms that describe nociceptive and neuropathic, such as “sore” and “stinging” [43]. However, the DLQI takes only two to three minutes to complete, requires no specialized equipment, and is free to administer [43].

## Therapeutic Management of Pain in HS

### Pharmacological Management of Nociceptive Pain: Stepwise Approach

Pain management in HS starts with disease control. Mild, localized pain is first treated with topical over-the-counter (OTC) analgesics such as lidocaine [44, 45]. Mild pain can also be treated with a topical OTC non-steroidal anti-inflammatory drug (NSAID) like diclofenac [19]. Corticosteroid injections with triamcinolone acetonide into HS nodules can reduce pain by reducing inflammation [46] if topical agents or steroid injections do not provide adequate pain relief. Patients can move up to using oral OTC acetaminophen or oral NSAIDS [47]. NSAIDs act by inhibiting cyclooxygenase enzymes COX-1 and COX-2. This inhibition results in decreased prostaglandin production and reduced acute inflammation. Finally, in patients with pain that is not controlled with first-line agents, short-term opioid treatment may be considered. Opioids act on G protein-coupled receptors on neurons in the central nervous system, causing hyperpolarization and a decrease in neurotransmitter release, which reduces pain perception [48]. Importantly, none of these agents halt HS progression; they provide only symptomatic relief. In other words, these agents cannot prevent the transition from acute inflammatory lesions in Hurley stage I to chronic sinus tracts and fibrosis in Hurley stages II and III [19, 41].

### Pharmacological Management of Neuropathic Pain

Neuropathic pain is challenging to treat because it arises from damage to or dysfunction of the somatosensory nervous system [49]. The preferred pharmacologic therapies for neuropathic pain are gabapentinoids, such as gabapentin and pregabalin, which bind to the α2δ subunit of presynaptic voltage-gated calcium channels, thereby reducing excessive neuronal excitation and thus neuropathic symptoms [50]. Serotonin and norepinephrine reuptake inhibitors (SNRIs), such as duloxetine and venlafaxine, have also been used to treat neuropathic pain in HS. These agents act at synapse sites and inhibit serotonin and norepinephrine transporters. Lower-tier agents for neuropathic pain include α_2_ agonists such as clonidine and dexmedetomidine. These uses would be off-label in treating HS neuropathic pain, and their efficacy may actually decrease in states of chronic neuropathic pain because they increase regulator of G protein signaling 4 (RGS4) activity [51–55]. Tricyclic antidepressants also inhibit the reuptake of serotonin and norepinephrine. Outside of ingested medications, topical agents such as lidocaine and capsaicin are also used [56]. Neuropathic pain can present differently in each patient, so combined efforts in prescribing the appropriate medication are vital. Again, none of these pharmacological therapies pauses HS progression, and none have been officially investigated in HS-specific clinical trials [49, 57].

### HiSCR 50 and Pharmacological Management of HS Using Biologics

The sole medications with proven efficacy in reducing the inflammatory component of HS are biologics and systemic antibiotics [19]. Surgical treatments are indicated for diseases that have progressed to significant structural damage due to tunneling and scarring [19, 46, 58]. HiSCR50 is the primary HS treatment outcome evaluated in most clinical trials. This tool indicates that a therapy has caused a clinically meaningful decline in the amount of inflammatory activity. HiSCR50 means there is at least a 50% decline in the combined number of abscesses and inflammatory nodules compared to baseline, with no expansion in tunneling or in the number of abscesses. Regardless of biologic use, however, advanced HS disease causes irreversible tissue damage [19, 46, 58]. Furthermore, biologic therapy cannot reverse already established tunnels and scarring [19, 46, 58].

There are three FDA-approved biologics, all of which are listed in Table 1. Adalimumab, an Igg1 monoclonal antibody that inhibits TNF-α, was the first FDA-approved biologic for moderate-to-severe HS in 2015 [59]. Adalimumab is the only HS biologic that has lower-cost biosimilars available as of July 2026. However, adalimumab biosimilars may have similar or slightly reduced efficacy [60]. A second biologic, secukinumab, an IgG1 monoclonal antibody that selectively inhibits IL-17 A, earned FDA approval in 2023 [61, 62]. Secukinumab was an important addition in HS biologic therapy because not all patients respond to adalimumab, others lose their response over time although it was effective earlier in treatment, and others experience adverse effects [63]. Compared to other inflammatory diseases, IL-17 F in HS is present in a much higher amount than IL-17 A, and both are implicated in the development of the deep-seated nodules seen in HS [31].

**Table 1:** Current FDA-approved biologic therapies for HS

Agent	Target	HiSCR50	Pain Instrument	Numerical Pain Result	FDA Approval Date	Source
Adalimumab (40 mg QW)	TNF-α	PIONEER I: 41.8% vs. 26.0% placebo (*p* = 0.003) PIONEER II: 58.9% vs. 27.6% placebo (*p* < 0.001)	^§^NRS30	*PIONEER I: 36.5% vs. 29.8% placebo (*p* = 0.369) PIONEER II: 53.3% vs. 22.9% placebo (*p* < 0.001)	2015	[59]
Bimekizumab (320 mg Q2W for first 16 wks, then 320 mg Q4W)	IL-17 A/IL-17 F	BE HEARD I: 48% vs. 29% placebo (*p* = 0.0060) BE HEARD II: 52% vs. 32% placebo (*p* = 0.0032)^a^	^§§^NRS30	^§§^BE HEARD I Q2W: 32% vs. 15% placebo (*p* = 0.041) *Q4W: 22% vs. 15% placebo (*p* = 0.37) BE HEARD II Q2W: 32% vs. 11% placebo (*p* = 0.010) Q4W: 29% vs. 11% (*p* = 0.028)	2024	[64]
Secukinumab (300 mg QW for first 5 wks, then 300 mg Q4W)	IL-17 A	SUNSHINE: 45% vs. 34% placebo (*p* = 0.0070) SUNRISE: 42% vs. 31% placebo (*p* = 0.015)	^§§§^NRS30	^§§§^Pooled SUNSHINE/SUNRISE, Q2W): 39% vs. 27% placebo (*p* = 0.0031) *Q4W: 36% vs. 27% placebo (*p* = 0.025)	2023	[62]

Additionally, dual inhibition of IL-17 A and IL-17 F had a greater effect in suppressing Th17-induced HS gene expression and neutrophil migration than inhibition of either alone [31]. This drove the development of bimekizumab. Bimekizumab, an IgG1 monoclonal antibody that selectively inhibits IL-17 A and IL-17 F, is the most recently FDA-approved biologic for HS, earning approval in 2024 [64, 65]. Bimekizumab more effectively targets the mediators that drive HS development [65]. Of the three FDA-approved biologics, bimekizumab appears to provide the largest absolute reduction in skin pain and a rapid onset of pain improvement as early as week 2 of treatment. However, direct comparison is difficult because the pain instruments and responder definitions differ.

Although the biologics in Table 1 have shown very high effectiveness in managing HS, responses to these treatments vary considerably among patients, highlighting the need for additional therapies that modulate or inhibit other inflammatory cascades. As knowledge of the immune pathways that drive HS continues to expand, new biologics and targeted therapies are being developed to address the complex, multifactorial inflammatory processes underlying HS pathogenesis. Janus kinase inhibitors (JAKi), which are currently used to treat a multitude of autoimmune diseases, have become encouraging therapeutic drug candidates for HS due to their ability to modify the intracellular signaling pathways that are implicated in immune system activation [11]. Povrocitinib and upadacitinib are both JAK inhibitors currently being investigated in Phase II clinical trials to treat HS [11, 66, 67]. JAKi’s may have interesting applications in pain medicine because they have both anti-nociceptive effects on peripheral sensory neurons and anti-inflammatory effects. These two JAKIs are not FDA-approved specifically for pain management. However, JAK1 inhibition prevents allodynia, driven by IFN-β, IFN-α, and IL-6 [68]. JAK inhibitors may have a more pronounced impact on pain than biologic DMARDs because of their direct effects on sensory nociceptive neurons and their anti-inflammatory properties. Still, there are no current clinical trials that directly compare biologic DMARDs and JAK inhibitors for pain head-to-head in HS [11]. This dual mechanism could make JAK a great choice for treating the high proportion of HS patients with mixed nociceptive and neuropathic pain [17, 66, 67]. Additionally, oral JAKis are a strong option for patients who feel uncomfortable with injections.

Similarly, while secukinumab and bimekizumab are FDA-approved for HS, additional IL-17 pathway inhibitors continue to be explored to improve disease control and address persistent symptoms in patients who have failed other biologic therapies [11]. Sonelokimab, a nanobody that inhibits IL-17 A and IL-17 F like bimekizumab, is currently being evaluated in Phase III clinical trials, with HiSCR75 at Week 16 as the primary outcome measure. However, sonelokimab is a nanobody (40 kDa), whereas bimekizumab is a monoclonal antibody (150 kDa) [64, 69]. Sonelokimab’s smaller size might allow it to penetrate deeper into the inflamed dermis and subcutaneous tissue, so it can suppress active inflammation at the source. Thus, sonelokimab might be better at reaching abscesses and tunnels that are difficult for large-molecule monoclonal antibodies to reach [70, 71]. Sonelokimab 120 mg Q4W had a higher HiSCR50 than adalimumab, bimekizumab, and secukinumab in a 2025 meta-analysis, but these drugs were not compared directly in a clinical trial [12]. Additionally, nanobodies are simpler and less expensive to produce because they can be made in yeast, which may make sonelokimab less expensive and thus more accessible than monoclonal antibodies, which require mammalian cell culture [72].

Other investigational biologics targeting inflammatory pathways involved in HS include spesolimab, an IL-36 receptor inhibitor, and lutikizumab, an IL-1α/β inhibitor, both of which are being studied as potential treatment options for HS [11, 73]. Keratinocytes produce IL-36 and induce IL-17, as well as neutrophil-attracting chemokines such as CXCL1 and G-CSF [74, 75]. IL-36 contributes to long-standing inflammatory pain [76]. Spesolimab showed a drastic improvement in the tunnels in HS, which is a key component that IL-17 inhibitors failed to address [73]. Spesolimab might be a great choice for patients with chronic and inflammatory pain as well as tunnel-predominant HS [73].

Lutikizumab showed promising results in patients that failed adalimumab: 59.5% achieved HiSCR50 vs. 35.0% placebo, and NRS30 was seen in 34.5% vs. 12.9% placebo [77]. IL-1β is expressed in HS lesions and drives a cascade that activates the matrix metalloproteinases that actually destroy tissue and cause formation of tunnels [32, 77]. These findings suggest that IL-1 blockade might offer clinically significant pain relief even in patients who failed TNF-α inhibition, making lutikizumab a promising biologic for adalimumab non-responders [77]. The adalimumab non-responder patient population has limited FDA-approved alternatives at this time [77].

RGRN-305 is an oral small-molecule therapy that targets heat shock protein 90 (HSP90) and offers another potential approach to reducing HS-related inflammation [78]. The advantage of RGRN-305 is that it disrupts multiple inflammatory pathways simultaneously, rather than targeting specific or dual cytokines or receptors as biologics do [78, 79]. In other words, RGRN-305 might be able to target the same pathways as JAKi’s, adalimumab, secukinumab, and lutikizimab all in one pill. HSP90 is a chaperone protein that ensures the proper functioning of NF-κB signaling and JAK-STAT signaling, among others [80]. NF-κB is the chief transcription factor that leads to the production of the cytokines TNF-α, IL-1β, IL-6, and IL-8 [80]. JAK-STAT signaling involves JAK1 and JAK2 [81].

However, despite the growing number of therapies under investigation, pain outcomes are not consistently reported in clinical trials [59, 62, 64, 66, 67, 70, 73, 77, 78]. Many new therapies are being investigated for HS because all current therapies fail to capture all drivers of the complex inflammation seen in HS. Only two emerging therapies, lutikizumab and RGRN-305, have reported numerical pain outcomes, which are listed in Table 2.

**Table 2:** Emerging therapies under investigation for HS

Agent	Type	Target	Published Phase	HiSCR50	Pain Instrument	Numerical Pain Result	Current Phase	Source
Lutikizumab (300 mg Q2W)	Biologic	IL-1α/β	Phase II RCT (*n* = 155)	59.5% vs. 35% placebo	NRS30^§^	34.5% (Q2W)/34.8% (QW) vs. 12.9% placebo	Phase III (ongoing in adults and adolescents)	[77]
RGRN-305 (250 mg QD)	Small molecule	HSP90	Phase II RCT (*n* = 15)	60% vs. 20% placebo	NRS30^§§^	60% vs. 40% placebo	Phase II completed (uncertain future)	[78]

Table 1. FDA-approved biologics for moderate-to-severe HS as of July 2026. Unless otherwise specified, HiSCR50 data reflect the Q2W (or weekly) dosing arm, which was the regimen that met statistical significance in each trial. NRS=Numeric rating scale; HiSCR 50 = Hidradenitis Suppurativa Clinical Response 50; wk=week; wks=weeks; HSSDD=Hidradenitis Suppurativa Symptom Daily Diary; QW=every week (weekly); Q2W=every 2 weeks; Q4W=every 4 weeks; pts=patients. Indicates that the included data were for patients who received bimekizumab every 2 weeks rather than every 4 weeks (both dosing intervals were included in the phase III clinical trials). *Indicates the data is *not* statistically significant. ^§^Pain response endpoint was defined as NRS30, with ≥ 30% reduction in NRS weekly worst skin pain score and ≥ 1 point reduction, and the endpoint was at week 12. ^§§^Pain response was defined as an improvement from baseline in HSSDD weekly worst skin pain score (NRS-equivalent) of ≥ 3 points among patients with a baseline score ≥ 3, and the endpoint was at week 16. ^§§§^Pain response was defined as an improvement from baseline of ≥ 30% reduction in weekly NRS pain score and ≥ 2-point reduction, and the endpoint was at week 16.

Table 2. Emerging therapies under investigation. Table only includes therapies with published numerical pain results. NRS=Numeric rating scale; HiSCR50 = Hidradenitis Suppurativa clinical response 50; QD=daily; QW=weekly; EOW=every other week. ^a^Indicates the current clinical trial phase as of July 2026. ^b^Indicates the dosing is through subcutaneous (SC) injection unless otherwise noted. ^§^Pain response was defined as an improvement from baseline of ≥ 30% reduction in weekly NRS pain score and ≥ 1-point reduction, and the endpoint was at week 16. ^§§^Indicates that the pain response was defined as ≥ 30% reduction in weekly NRS pain score and ≥ 1-point absolute reduction.

### Interventional and Procedural Approaches

Nociceptive and neuropathic pain both comprise the long-standing pain observed in HS, but currently there is a paucity of interventional pain studies specific to this population. Currently, there are no American Academy of Dermatology (AAD) guidelines for nerve blocks, spinal cord stimulation (SCS), dorsal root ganglion (DRG) stimulation, or transcutaneous electrical nerve stimulation (TENS), highlighting a crucial need for studies that assess the effectiveness of these procedures in HS patients with long-standing pain, particularly when conventional pharmacologic management are insufficient [45].

Local anesthetic is typically injected into HS lesions and the surrounding areas in procedural settings of incision and drainage, deroofing sinus tracts, and excision of affected skin [71]. However, this has led to inadequate anesthesia, as the areas of inflammation and fibrosis have impaired the efficacy of local anesthetic [71]. Regional nerve blocks can be utilized in these procedures for more reliable pain control [82]. For lesions in the axillary region, a case report describes a modified pectoral nerve (PECS) block II, with the injection between the pectoralis minor and serratus anterior muscles, that can target the lateral branches of the intercostal nerves providing sensory innervation to the axilla [82] In this case, the modified PECS block II showed effective pain relief without requiring additional local anesthetics [82]. Building on the potential use of nerve blocks in HS patients, ilioinguinal and iliohypogastric nerve blocks are routinely utilized in inguinal and groin procedures. In contrast, pudendal nerve blocks are used in vulval and groin surgery [83, 84]. In an observational study, patients undergoing ilioinguinal and pudendal nerve blocks for vulval and groin surgery demonstrated high pain control with 94% of patients reporting low Visual Analogue Scale (VAS) scores of 0–3 on postoperative day one [83].

In patients whose pain persists despite pharmacologic therapies and surgical procedures, neuromodulation may be a viable mode of pain management escalation. SCS modulates nociceptive processing through epidural electrodes implanted near the dorsal columns of the spinal cord. A 2026 meta-analysis of 16 randomized control trials included 1,479 participants with an array of chronic pain conditions who demonstrated a considerable decrease in pain and improved quality of life with SCS versus standard medical management, with low to moderate certainty of evidence [85]. SCS has not been recommended for HS yet, but it can benefit HS patients with severe, chronic HS-related pain if the pain involves a wide dermatomal distribution. However, there are risks involved with epidural electrode implantation, including infection and shifting of electrodes post-placement, which must be taken into account in a population that is predisposed to infections and wound complications [86].

SCS provides neuromodulation across a diffuse anatomical area, whereas DRG stimulation modulates specific sensory neurons within the DRG for pain alleviation only in certain dermatomal regions. The FDA has authorized DRG stimulation for pain in the T10-S2 dermatomes, including the inguinal, perineal, and gluteal regions commonly impacted by HS [87, 88]. For complex regional pain syndrome, the ACCURATE trial showed a considerable decrease in pain with DRG stimulation versus SCS, with ≥ 50% pain relief in 74% versus 51% of patients at three months, respectively. DRG has less variability in electrode placement, which can lead to more consistent, targeted pain relief than SCS. DRG stimulation may be advantageous for HS patients. Still, studies have not been conducted in this specific population, demonstrating an area of future research in these patients with chronic pelvic and inguinal pain.

TENS is a noninvasive neuromodulation modality that electrically stimulates the site of pain through cutaneous electrodes, activating Aβ fibers to decrease nociceptive transmission [89, 90]. TENS can avoid the procedural risks associated with SCS and DRG stimulation, but there may be practical limitations of cutaneous electrode usage on inflamed or scarred skin. Nevertheless, TENS may be used in HS patients with long-standing pain around active sites of inflammation, as electrodes can be placed adjacent to, but not in, the active lesions to reduce nociceptive pain [78, 79]. Thus, TENS can serve as a low-risk adjunctive therapy for long-standing pain adjacent to active HS lesions [90, 91].

## Special Populations and Considerations

Pharmacologic management and interventional procedures are only impactful if patients have access to them. The literature demonstrates considerable disparities in pain management for HS patients. The prevalence of HS in Black Americans is more than double that of the White population [19, 46]. After controlling for age, sex, MI, tobacco use, and insurance type, a study by Ulschmid et al. found that Black patients with HS were 2.8 times more likely to have progressed to the most severe form of the disease, Hurley stage III, than White patients [92]. Black patients had 2.86 times the risk of visiting the emergency department (ED) for HS-related symptoms, 2.25 times the risk for hospitalization, and 1.61 times the risk of undergoing a surgical procedure versus White patients [92]. Hispanic patients with HS also present to the ED with higher disease severity and higher healthcare usage [93].

There are several obstacles to pain control in HS patients that compound the disparities of disease burden seen. A worldwide study of 890 patients with HS found that 38.4% were never prescribed pain medication [84]. Barriers included healthcare providers not asking about pain and reluctance to treat the pain due to limited knowledge regarding HS [94]. A qualitative study of Latine patients identified further barriers: inadequate wound care discussion and guidance from physicians, the burden of creating a self-directed wound care on the patient without adequate support, and a lack of insurance coverage for wound care supplies [85]. Additionally, perceived discrimination from healthcare providers may create a fear of stigma, which leads to avoidance of care, especially when patients attempt to self-manage their pain during HS flares with unsafe medication dosages because they are unable to access their healthcare provider promptly [37, 95]. Language barriers continue to impede patient-centered care, as patients seek out a physician who speaks their language or use basic English proficiency to communicate instead of using a translator service [95].

In treatment disparities, the prescribed medication is a factor. Black patients with HS are more likely to be prescribed antibiotics and immunosuppressive agents but less likely to receive small molecule inhibitors, suggesting limited access to newer therapies in this population [96]. When prescribing opioids, Black patients received an average annual dose that is 36% lower than doses prescribed to White patients, a pattern that persisted across 91% of 310 healthcare systems studied [97].

Opioid use disorder is a risk that may develop in HS patients who may have severe chronic pain, psychological comorbidities, and limited access to comprehensive care. A population-based analysis found the prevalence of substance use disorder (SUD) in individuals with HS at 4.0%, compared to 2.0% in individuals without HS, with opioid misuse accounting for 32.7% of SUD in this population [98]. There is an increased risk of HS patients frequently visiting the ED for pain management: 48.7% of patients will fill the opioid prescription from their ED visit within one week [99]. Receiving an opioid prescription from the ED is associated with increased odds of return to the ED within 30 days (odds ratio (OR) 1.67) and 180 days (OR 1.48) but decreased odds of following up with the dermatologist within 30 days (OR 0.78) and 180 days (0.81), where opioids manage temporary pain relief but nothing done to address the underlying HS flare [99].

## Discussion

Neuropathic pain contributes significantly to the quality-of-life impairment in HS. The pain can persist beyond active lesions in the absence of flares, hindering sleep, daily movement, and mood, underscoring the need for recognition to provide comprehensive patient care [37]. Among the FDA-approved biologics, there is a lack of stratification between neuropathic versus non-neuropathic pain profiles [59, 64]. Additionally, there is no pain assessment method used in common across the trials for each biologic, which impedes direct comparison for pain management [100]. None of the HS clinical trials for adalimumab, bimekizumab, or secukinumab have differentiated, measured, or reported neuropathic pain as a distinct outcome [59, 62, 64]. All three phase III clinical trials for the FDA-approved biologics used NRS or a modified version of the NRS (HSSDD) without neuropathic-specific assessments such as PainDETECT and QST [22, 45]. The most rigorous biologics trials fail to capture pain as a multidimensional outcome, leaving the neuropathic component uncharacterized and unaddressed [17, 59, 62, 64]. Even bimekizumab, which provides the largest absolute reduction in skin pain, has not been evaluated using pain instruments that could distinguish whether this improvement is due to nociceptive pain relief, neuropathic pain relief, or both [64]. Current evidence suggests that biologics in HS are the most effective in reducing pain driven by inflammation, or nociceptive pain [19]. Nociceptive pain in HS is caused by the active abscesses, tunnels, and nodules [94]. However, not all pain in HS is driven by inflammation [17, 33]. To date, no clinical trials have evaluated the effect of biologics on neuropathic pain in HS.

To this end, the available evidence for biologics treating neuropathic pain is primarily indirect and mechanistic. One study that approximated QST to PainDETECT, which is more feasible for routine clinical use as a brief questionnaire, found that 64% of HS patients felt allodynia or paradoxical thermal sensations, hallmarks of neuropathic pain [39]. Biologics may already be treating neuropathic pain through inflammatory mediators-mediated pathways. TNF-α is upregulated in DRGs after nerve injury and promotes pain by increasing the current through the voltage-gated sodium channel [101–103]. IL-17 A activates signaling pathways in spinal neurons to regulate neuropathic pain [104, 105]. IL-17 F has been shown to increase sodium currents in DRG neurons [106, 107]. Collectively, these findings suggest that the same cytokines responsible for inflammatory HS lesions may also sensitize peripheral nerves, providing a plausible mechanistic link between biologics and neuropathic pain relief that has not been tested.

Neuropathic pain might therefore be treated using biologics, especially bimekizumab, but no meaningful conclusions can be drawn at this time. Future clinical trials for novel biologics should use neuropathic pain instruments as well as nociceptive pain instruments to stratify patients’ pain and determine whether adjunctive neuropathic pain management is necessary [108, 109]. While there are more novel biologics for HS than those listed in this narrative review, the number included in this paper was limited by the number of clinical trials that reported pain measures. There are no specific HS pain studies in the literature, which is a huge unmet need in HS research [45]. Recommendations for HS pain treatment are based on patient preference and adapted from general pain guidelines [19, 45].

This gap is further compounded by staging limitations. Biologics are not approved for Hurley Stage I, even though Hurley Stage I patients can still experience neuropathic pain. Neuropathic pain positively correlates with IHS4 (International Hidradenitis Suppurativa Severity Score System) and not with Hurley Stage [110]. In other words, patients with Hurley stage I may have more neuropathic pain than stage III patients. No biologics are FDA-approved for Hurley stage I, however. The 2017 IHS4 may be a better indicator of suitability for a biologic than the traditionally used Hurley staging system [34]. Biologics may be the best choice for neuropathic pain in all stages because they directly inhibit the cytokines that contribute to its pathogenesis. Clinicians should evaluate whether biologics are appropriate for patients with Hurley stage I. Prescribing any biologic in this patient population would be off-label, and clinicians should ensure patients have informed consent. However, no conclusion can be made at this time.

This raises the possibility of a “window of opportunity” for neuropathic pain prevention in HS [111]. In HS, the “window of opportunity” is the time from symptom onset when starting targeted therapies, especially biologics, that yields the best disease outcomes. This is because biologics prevent the transition from inflammatory disease (reversible) to structural damage (irreversible) [111]. The repeated inflammation in early HS is the most responsive to biologic treatment, based on current research [59, 62, 64]. The tunneling and scarring in late HS cause physical damage to peripheral nerve fibers, which leads to neuropathic pain in HS [22]. Because there is no universally agreed upon definition of moderate disease, clinicians should consider prescribing biologics when patients have at least one inflamed skin tunnel or at least four inflammatory abscesses or nodules in at least two different anatomical areas [111]. In 2026, the European Hidradenitis Suppurativa Foundation Delphi consensus consisting of 54 HS experts similarly recommended that biologics or small molecules should be first-line for patients with rapidly progressing disease, contraindications to antibiotics, and severe disease, among other factors [112]. These consensus criteria for starting biologics early in the disease align with the proposed window of opportunity for preventing neuropathic pain, even though pain outcomes were not explicitly included in the Delphi criteria [112].

Beyond pharmacologic gaps, there are currently no American Academy of Dermatology (AAD) guidelines for nerve blocks, spinal cord stimulation, dorsal root ganglion stimulation, or transcutaneous electrical nerve stimulation in HS, underscoring the critical need for studies evaluating these procedures. Moreover, there is no validated, uniform pain endpoint across pivotal HS trials. This makes cross-drug efficacy comparisons for pain extremely difficult. Given its burden and effects on all aspects of a patient’s life, pain should be a secondary endpoint in HS clinical trials [113]. HS is ultimately a chronic disease that requires a multidisciplinary team of dermatologists, anesthesiologists, and psychiatrists for management [57].

These gaps do not fall evenly across the patient population. The literature currently shows that racial and ethnic disparities in HS exist beyond differences in disease prevalence, including inequities in pain management and access to comprehensive management. Black and Hispanic patients may experience more severe disease due to delays in diagnosis, barriers in accessing specialty care, and difficulty affording wound care supplies [19, 37, 46, 93–95]. These aggregating factors may contribute to the undertreatment of pain, which may lead to frequent reliance on EDs and frequent opioid exposure [99]. Furthermore, insurance-related barriers worsen this issue. For example, Medicaid requires stepwise therapy in which, depending on the state, patients must fail trials of specific antibiotics (clindamycin, minocycline, doxycycline, and rifampin) lasting at least 3 months [114, 115]. For Louisiana Medicaid, patients must be explicitly diagnosed with Hurley stage II or III to qualify for adalimumab [116]. Neuropathic pain management is important because of the gaps in diagnoses and treatment currently, and new treatments are available as alternative or additional treatments for neuropathic pain [117–119]. With the increased risk of opioid use disorder in HS patients, healthcare systems also need to provide equitable access to care, implement routine assessments for pain, foster culturally competent communication, and optimize therapy for HS to reduce pain burden and improve outcomes in all patient populations.

## Conclusion

HS is a lifelong, irritating disease of the skin that predominantly affects women and carries a significant psychosocial burden. Pain is the symptom that causes the most distress for patients. Neuropathic pain is an underrecognized but clinically significant contributor to this burden. In HS, this subtype of pain arises from scarring, repeated abscess formation, and tunneling in later disease, all of which damage nearby sensory neurons. Despite the high proportion of HS patients with neuropathic pain, no clinical trial as of July 2026 has graded HS patients by pain subtype or utilized neuropathic pain screening tools such as PainDETECT. All three FDA-approved biologics (adalimumab, bimekizumab, and secukinumab) failed to differentiate, measure, or report neuropathic pain as a distinct outcome because they relied on the NRS. This instrument cannot distinguish between nociceptive and neuropathic pain. Because of this, the question of whether biologics treat the neuropathic pain in HS is still unanswered. The possibility of biologics treating neuropathic pain is strengthened only by indirect mechanistic evidence. Because biologics target cytokines that also drive neuropathic pain, it is plausible that these biologic therapies may treat neuropathic pain as well. The lack of pain stratification in clinical trials has direct clinical consequences. Clinicians currently lack a method of determining which patients’ pain will respond to biologic therapy alone, preventing clinicians from determining which biologic would be the best fit for a patient based on the pain they are experiencing.

This pain measurement gap is further compounded by racial and ethnic disparities in HS diagnosis and disease severity, as well as insurance-related barriers. Louisiana Medicaid, for example, requires patients to fail a three-month trial of antibiotics and have explicit documentation of Hurley stage II or III for adalimumab approval. These requirements delay access to disease-modifying therapy for patients who may already be facing barriers to specialty care and appropriate diagnosis. A separate but related issue is that the FDA currently uses the Hurley staging system to determine which patient population can access disease-modifying biologics. However, neuropathic pain can be present in all Hurley stages. Neuropathic pain has a stronger positive correlation with the 2017 IHS4 than the 1989 Hurley staging system. Patients with Hurley stage I disease are not eligible for FDA-approved biologics, but they may still experience neuropathic pain that current treatment step-up ladders do not manage. Severe pain at Hurley stage I may itself be an indicator of rapidly progressing disease, which may warrant reconsideration of current staging-based eligibility criteria for patients to receive biologic therapy. Thus, there is a potential window of opportunity where starting biologics earlier in the disease can prevent the transition from reversible inflammatory pain to irreversible nerve and tissue damage.

To address this unmet need, future clinical trials should incorporate neuropathic pain-specific instruments alongside standard HiSCR outcomes. Pain should be formally established as a secondary endpoint in HS clinical trials due to its burden and effects on all aspects of a patient’s life. For now, clinicians should consider routinely screening HS patients for neuropathic pain using the brief PainDETECT survey to fill this gap in the clinic. Clinicians should consider first-line treatment for neuropathic pain, gabapentin and pregabalin, in patients who are found to have a high clinical suspicion of neuropathic pain. Pain measurement should keep pace with the rate of treatment innovation to comprehensively care for patients with HS.

## Key References

- Kimball B, et al. Two Phase 3 Trials of Adalimumab for Hidradenitis Suppurativa. N Engl J Med. 2016;375(5):422–34. https://doi.org/10.1056/NEJMoa1504370.
  - ○ This reference highlights the results from PIONEER I and PIONEER II, two phase III clinical trials for adalimumab, one of three currently FDA-approved biologics for the treatment of moderate-to-severe HS. This article details HiSCR50 and pain outcome endpoints.
- Kimball AB, et al. Secukinumab in moderate-to-severe hidradenitis suppurativa (SUNSHINE and SUNRISE): week 16 and week 52 results of two identical, multicentre, randomized, placebo-controlled, double-blind phase 3 trials. Lancet. 2023;401(10378):747–61. https://doi.org/10.1016/S0140-6736(23)00022-3.
  - ○ This reference highlights the results from SUNSHINE and SUNRISE, two phase III clinical trials for secukinumab, the second of three currently FDA-approved biologics for the treatment of moderate-to-severe HS. This article details HiSCR50 and pain outcome endpoints.
- Kimball AB, et al. Efficacy and safety of bimekizumab in patients with moderate-to-severe hidradenitis suppurativa (BE HEARD I and BE HEARD II): two 48-week, randomised, double-blind, placebo-controlled, multicentre phase 3 trials. Lancet. 2024;403(10443):2504–19. https://doi.org/10.1016/S0140-6736(24)00101-6.
  - ○ This reference highlights the results from BE HEARD I and BE HEARD II, two phase III clinical trials for bimekizumab, the most recently FDA-approved biologic for the treatment of moderate-to-severe HS. This article details HiSCR50 and pain outcome endpoints.

## Data Availability

No datasets were generated or analysed during the current study.

## References

[1] Wolk K, Join-Lambert O, Sabat R. Aetiology and pathogenesis of hidradenitis suppurativa. Br J Dermatol. 2020;183:999–1010. 10.1111/bjd.19556. 33048349

[2] Nguyen TV, Damiani G, Orenstein LaV, Hamzavi I, Jemec GB. Hidradenitis suppurativa: an update on epidemiology, phenotypes, diagnosis, pathogenesis, comorbidities and quality of life. J Eur Acad Dermatol Venereol JEADV. 2021;35:50–61. 10.1111/jdv.16677. 32460374

[3] Peacker BL, Ly L, Hwang JC, Gravel-Pucillo K, Zheng C, Wheless LE, et al. Multiomics analyses prioritize disease genes and pathways in hidradenitis suppurativa. J Invest Dermatol. 2026;146:1807–e18152. 10.1016/j.jid.2025.12.025. 41548865

[4] McCarthy S. Hidradenitis Suppurativa. Annu Rev Med. 2025;76:69–80. 10.1146/annurev-med-051223-031234. 39869430

[5] Look-Why S, Kirimlishvili S, Porter M, Kimball A. Hidradenitis Suppurativa Epidemiology - ClinicalKey. Dermatol Clin. 2025;43:145–54. 40023617 10.1016/j.det.2024.12.001

[6] Midgette B, Garg A. Epidemiology of hidradenitis suppurativa and its comorbid conditions. J Am Acad Dermatol. 2024;91:S3–7. 10.1016/j.jaad.2024.09.013. 39626996

[7] Tsentemeidou A, Sotiriou E, Ioannides D, Vakirlis E. Hidradenitis suppurativa-related expenditure, a call for awareness: systematic review of literature. JDDG J Dtsch Dermatol Ges. 2022;20:1061–72. 10.1111/ddg.14796. 35821567

[8] Saunte DML, Jemec GBE. Hidradenitis Suppurativa: Advances in Diagnosis and Treatment. JAMA. 2017;318:2019–32. 10.1001/jama.2017.16691. 29183082

[9] Shukla R, Karagaiah P, Patil A, Farnbach K, Ortega-Loayza AG, Tzellos T, et al. Surgical treatment in hidradenitis suppurativa. J Clin Med. 2022;11:2311. 10.3390/jcm11092311. 35566438 PMC9101712

[10] Mendes-Bastos P, Benhadou F, Venturini M, Molina-Levya A, Thomas N, Alarcon I, et al. Biologic drugs in hidradenitis suppurativa: what does the GP have to know? A narrative review. Front Med. 2024;11:1403455. 10.3389/fmed.2024.1403455. PMC11261743 39040895

[11] Garg A, Hsiao J, Porter ML, Shi V. Current treatments and future directions for hidradenitis suppurativa: a narrative review of completed and ongoing phase 3 clinical trials of biologic therapies. Dermatol Ther. 2025;15:2361–77. 10.1007/s13555-025-01487-y. PMC12354412 40681937

[12] Garg A, Cohn E, Midgette B, Frasier K, Strunk A. Efficacy and Safety of Medical Interventions for Moderate to Severe Hidradenitis Suppurativa: A Living Systematic Review and Network Meta-Analysis. JAMA Dermatol. 2025;161:931–40. 10.1001/jamadermatol.2025.1976. 40601333 PMC12224058

[13] Chen SX, Wafae BG, de O, Look-Why S, Otto T, Snyder C, Sweet W, et al. Therapeutic drug monitoring of adalimumab in hidradenitis suppurativa: a prospective observational study. Front Immunol. 2026;17:1760206. 10.3389/fimmu.2026.1760206. 41727443 PMC12917758

[14] Diaz MJ, Aflatooni S, Abdi P, Li R, Anthony MR, Neelam S, et al. Hidradenitis suppurativa: molecular etiology, pathophysiology, and management—a systematic review. Curr Issues Mol Biol. 2023;45:4400–15. 10.3390/cimb45050280. 37232749 PMC10217408

[15] Krajewski PK, Matusiak Ł, von Stebut E, Schultheis M, Kirschner U, Nikolakis G, et al. Pain in Hidradenitis Suppurativa: A Cross-sectional Study of 1,795 Patients. Acta Derm Venereol. 2021;101:adv00364. 10.2340/00015555-3724. 33320274 PMC9309859

[16] Nielsen RM, Lindsø Andersen P, Sigsgaard V, Theut Riis P, Jemec GB. Pain perception in patients with hidradenitis suppurativa. Br J Dermatol. 2020;182:166–74. 10.1111/bjd.17935. 30919930

[17] Garcovich S, Muratori S, Moltrasio C, Buscemi AA, Giovanardi G, Malvaso D, et al. Prevalence of neuropathic pain and related characteristics in hidradenitis suppurativa: a cross-sectional study. J Clin Med. 2020;9:4046. 10.3390/jcm9124046. 33333779 PMC7765202

[18] Kendroud S, Fitzgerald LA, Murray IV, Hanna A. Physiology, Nociceptive Pathways. In: StatPearls. Treasure Island (FL): StatPearls Publishing; 2026. 29262164

[19] Sabat R, Alavi A, Wolk K, Wortsman X, McGrath B, Garg A, et al. Hidradenitis suppurativa. Lancet. 2025;405:420–38. 10.1016/S0140-6736(24)02475-9. 39862870

[20] Woolf CJ. What is this thing called pain? J Clin Invest. 2010;120:3742–4. 10.1172/JCI45178. 21041955 PMC2965006

[21] Nicholson B. Differential diagnosis: nociceptive and neuropathic pain. Am J Manag Care. 2006;12:S256–262. 16774457

[22] Alsouhibani A, Speck P, Cole EF, Mustin DE, Li Y, Barron JR, et al. Quantitative sensory testing to characterize sensory changes in hidradenitis suppurativa skin lesions. JAMA Dermatol. 2023;159:1102–11. 10.1001/jamadermatol.2023.3243. 37702999 PMC10500434

[23] Hartrick CT, Kovan JP, Shapiro S. The numeric rating scale for clinical pain measurement: a ratio measure? Pain Pract. 2003;3:310–6. 10.1111/j.1530-7085.2003.03034.x. 17166126

[24] Melchor J, Prajapati S, Pichardo RO, Feldman SR. Cytokine-mediated molecular pathophysiology of hidradenitis suppurativa: a narrative review. Skin Appendage Disord. 2024;10:172–9. 10.1159/000536268. 38835710 PMC11147516

[25] Binshtok AM, Wang H, Zimmermann K, Amaya F, Vardeh D, Shi L, et al. Nociceptors Are Interleukin-1β Sensors. J Neurosci. 2008;28:14062–73. 10.1523/JNEUROSCI.3795-08.2008. 19109489 PMC2690713

[26] Tang J, Zhao S, Shi H, Li X, Ran L, Cao J, et al. Effects on peripheral and central nervous system of key inflammatory intercellular signalling peptides and proteins in psoriasis. Exp Dermatol. 2024;33:e15104. 10.1111/exd.15104. 38794817

[27] Lowe MM, Naik HB, Clancy S, Pauli M, Smith KM, Bi Y, et al. Immunopathogenesis of hidradenitis suppurativa and response to anti–TNF-α therapy. JCI Insight. 2022;5. 10.1172/jci.insight.139932. PMC7566733 32841223

[28] García-Domínguez M. The role of TNF-α in neuropathic pain: an immunotherapeutic perspective. Life. 2025;15:785. 10.3390/life15050785. 40430212 PMC12113436

[29] Sorge RE, Si Y, Norian LA, Guha A, Moore GE, Nabors LB, et al. Inhibition of the RNA Regulator HuR by SRI-42127 Attenuates Neuropathic Pain After Nerve Injury Through Suppression of Neuroinflammatory Responses. Neurotherapeutics. 2022;19:1649–61. 10.1007/s13311-022-01278-9. 35864415 PMC9606176

[30] Salvemini D, Kim SF, Mollace V. Reciprocal regulation of the nitric oxide and cyclooxygenase pathway in pathophysiology: relevance and clinical implications. Am J Physiol - Regul Integr Comp Physiol. 2013;304:R473–87. 10.1152/ajpregu.00355.2012. 23389111 PMC4422342

[31] Rastrick J, Edwards H, Ferecskó AS, Le Friec G, Manghera A, Page M, et al. The roles of interleukin (IL)-17A and IL-17F in hidradenitis suppurativa pathogenesis: evidence from human in vitro preclinical experiments and clinical samples. Br J Dermatol. 2025;192:660–71. 10.1093/bjd/ljae442. 39531733

[32] Witte-Händel E, Wolk K, Tsaousi A, Irmer ML, Mößner R, Shomroni O, et al. The IL-1 pathway is hyperactive in hidradenitis suppurativa and contributes to skin infiltration and destruction. J Invest Dermatol. 2019;139:1294–305. 10.1016/j.jid.2018.11.018. 30528824

[33] Aarts P, Aitken JJ, van Straalen KR. Prevalence of Central Sensitization in Patients With Hidradenitis Suppurativa. JAMA Dermatol. 2021;157:1–5. 10.1001/jamadermatol.2021.2918. PMC8374732 34406352

[34] Aarts P, Dudink K, Vossen ARJV, van Straalen KR, Ardon CB, Prens EP, et al. Clinical implementation of biologics and small molecules in the treatment of hidradenitis suppurativa. Drugs. 2021;81:1397–410. 10.1007/s40265-021-01566-2. 34283386 PMC8352818

[35] 41 - Quantitative Sensory Testing. and Skin Biopsies Suggest Pain in Hidradenitis Suppurativa may be both Inflammatory and Neuropathic - Google Search n.d. https://www.jpain.org/article/S1526-5900(25)00915-0/fulltext (accessed July 8, 2026).

[36] Wright S, Strunk A, Garg A. Prevalence of depression among children, adolescents, and adults with hidradenitis suppurativa. J Am Acad Dermatol. 2022;86:55–60. 10.1016/j.jaad.2021.06.843. 34144081

[37] Orenstein LAV, Salame N, Siira MR, Urbanski M, Flowers NI, Echuri H, et al. Pain Experiences among those Living with Hidradenitis Suppurativa: A Qualitative Study. Br J Dermatol. 2023;188:41–51. 10.1093/bjd/ljac018. 36689519 PMC12536937

[38] Freynhagen R, Baron R, Gockel U, Tölle TR. PainDETECT: a new screening questionnaire to identify neuropathic components in patients with back pain. Curr Med Res Opin. 2006;22:1911–20. 10.1185/030079906X132488. 17022849

[39] Speck PJ, Alsouhibani A, Mustin DE, Cole EF, Harper DE, Orenstein LAV. Correlation between PainDETECT Questionnaire and quantitative sensory testing for the detection of neuropathic pain in hidradenitis suppurativa. Dermatology. 2024;240:152–5. 10.1159/000533262. 37494917

[40] Rolke R, Baron R, Maier C, Tölle TR, Treede -DR, Beyer A, et al. Quantitative sensory testing in the German Research Network on Neuropathic Pain (DFNS): standardized protocol and reference values. Pain. 2006;123:231–43. 10.1016/j.pain.2006.01.041. 16697110

[41] Savage KT, Singh V, Patel ZS, Yannuzzi CA, McKenzie-Brown AM, Lowes MA, et al. Pain management in hidradenitis suppurativa and a proposed treatment algorithm. J Am Acad Dermatol. 2021;85:187–99. 10.1016/j.jaad.2020.09.039. 32950543 PMC8176324

[42] Mücke M, Cuhls H, Radbruch L, Baron R, Maier C, Tölle T, et al. Quantitative sensory testing (QST). Engl version Schmerz. 2021;35:153–60. 10.1007/s00482-015-0093-2. 26826097

[43] Finlay Ay, Khan Gk. Dermatology Life Quality Index (DLQI)—a simple practical measure for routine clinical use. Clin Exp Dermatol. 1994;19:210–6. 10.1111/j.1365-2230.1994.tb01167.x. 8033378

[44] Alikhan A, Lynch PJ, Eisen DB. Hidradenitis suppurativa: A comprehensive review. J Am Acad Dermatol. 2009;60:539–61. 10.1016/j.jaad.2008.11.911. 19293006

[45] Alikhan A, Sayed C, Alavi A, Alhusayen R, Brassard A, Burkhart C, et al. North American clinical management guidelines for hidradenitis suppurativa: A publication from the United States and Canadian Hidradenitis Suppurativa Foundations: Part I: Diagnosis, evaluation, and the use of complementary and procedural management. J Am Acad Dermatol. 2019;81:76–90. 10.1016/j.jaad.2019.02.067. 30872156 PMC9131894

[46] Hamad J, McCormick BJ, Sayed CJ, Paci K, Overton M, Daubert T, et al. Multidisciplinary Update on Genital Hidradenitis Suppurativa: A Review. JAMA Surg. 2020;155:970. 10.1001/jamasurg.2020.2611. 32838413

[47] Scheinfeld N. Treatment of hidradenitis supprurativa associated pain with nonsteroidal anti-inflammatory drugs, acetaminophen, celecoxib, gabapentin, pegabalin, duloxetine, and venlafaxine. Dermatol Online J. 2013;19:20616. 24314785

[48] Pathan H, Williams J. Basic opioid pharmacology: an update. Br J Pain. 2012;6:11–6. 10.1177/2049463712438493. 26516461 PMC4590096

[49] Soliman N, Moisset X, Ferraro MC, de Andrade DC, Baron R, Belton J, et al. Pharmacotherapy and non-invasive neuromodulation for neuropathic pain: a systematic review and meta-analysis. Lancet Neurol. 2025;24:413–28. 10.1016/S1474-4422(25)00068-7. 40252663

[50] Fornasari D. Pharmacotherapy for Neuropathic Pain: A Review. Pain Ther. 2017;6:25–33. 10.1007/s40122-017-0091-4. 29178034 PMC5701897

[51] Yoon S-Y, Roh D-H, Yeo J-H, Woo J, Han SH, Kim K-S. Analgesic efficacy of α2 adrenergic receptor agonists depends on the chronic state of neuropathic pain: role of regulator of G protein signaling 4. Neuroscience. 2021;455:177–94. 10.1016/j.neuroscience.2020.12.021. 33359660

[52] Feeney JM, Noble RK, Bautista TB, Patel M, Abd-Elsayed A, Ahmadzadeh S, et al. Clinical Efficacy and Risks of Clonidine Infusion for Sedation and Analgesia: A Narrative Review. Curr Pain Headache Rep. 2026;30:70. 10.1007/s11916-026-01506-3. 42144510

[53] Feeney JM, Duet SJ, Jones CB, Baffi AJ, Rayes Elmalakh S, Bembenick KN, et al. Safety and efficacy of dexmedetomidine as an adjuvant in epidural anesthesia for labor analgesia: a narrative review. Med Sci. 2026;14:144. 10.3390/medsci14010144. PMC13027594 41892860

[54] Rekatsina M, Theodosopoulou P, Staikou C. Perioperative dexmedetomidine or lidocaine infusion for the prevention of chronic postoperative and neuropathic pain after gynecological surgery: a randomized, placebo-controlled, double-blind study. Pain Ther. 2022;11:529–43. 10.1007/s40122-022-00361-5. 35167059 PMC9098708

[55] Eisenach JC, DuPen S, Dubois M, Miguel R, Allin D, Epidural Clonidine Study Group, et al. Epidural clonidine analgesia for intractable cancer pain. Pain. 1995;61:391–9. 10.1016/0304-3959(94)00209-W. 7478682

[56] Fattori V, Hohmann MSN, Rossaneis AC, Pinho-Ribeiro FA, Verri WA. Capsaicin: current understanding of its mechanisms and therapy of pain and other pre-clinical and clinical uses. Molecules. 2016;21:844. 10.3390/molecules21070844. 27367653 PMC6273101

[57] Jeha GM, Kodumudi V, O’Quinn MC, Luckett KO, Dickerson TG, Kaye RJ, et al. Management of Acute and Chronic Pain Associated With Hidradenitis Suppurativa: A Comprehensive Review of Pharmacologic and Therapeutic Considerations in Clinical Practice. Cutis. 2021;108:281–6. 10.12788/cutis.0383. 35100536

[58] Krueger JG, Frew J, Jemec GBE, Kimball AB, Kirby B, Bechara FG, et al. Hidradenitis suppurativa: new insights into disease mechanisms and an evolving treatment landscape. Br J Dermatol. 2024;190:149–62. 10.1093/bjd/ljad345. 37715694

[59] Kimball AB, Okun MM, Williams DA, Gottlieb AB, Papp KA, Zouboulis CC, et al. Two Phase 3 Trials of Adalimumab for Hidradenitis Suppurativa. N Engl J Med. 2016;375:422–34. 10.1056/NEJMoa1504370. 27518661

[60] Burlando M, Fabbrocini G, Marasca C, Dapavo P, Chiricozzi A, Malvaso D, et al. Adalimumab Originator vs. Biosimilar in Hidradenitis Suppurativa: A Multicentric Retrospective Study. Biomedicines. 2022;10:2522. 10.3390/biomedicines10102522. 36289787 PMC9599334

[61] Fernandez-Crehuet P, Haselgruber S, Padial-Gomez A, Vasquez-Chinchay F, Fernandez-Ballesteros MD, López-Riquelme I, et al. Short-Term Effectiveness, Safety, and Potential Predictors of Response of Secukinumab in Patients with Severe Hidradenitis Suppurativa Refractory to Biologic Therapy: A Multicenter Observational Retrospective Study. Dermatol Ther. 2023;13:1029–38. 10.1007/s13555-023-00906-2. PMC10060482 36892752

[62] Kimball AB, Jemec GBE, Alavi A, Reguiai Z, Gottlieb AB, Bechara FG, et al. Secukinumab in moderate-to-severe hidradenitis suppurativa (SUNSHINE and SUNRISE): week 16 and week 52 results of two identical, multicentre, randomised, placebo-controlled, double-blind phase 3 trials. Lancet. 2023;401:747–61. 10.1016/S0140-6736(23)00022-3. 36746171

[63] Martora F, Marasca C, Cacciapuoti S, Fariello F, Potestio L, Battista T, et al. Secukinumab in hidradenitis suppurativa patients who failed adalimumab: a 52-week real-life study. Clin Cosmet Investig Dermatol. 2024;17:159–66. 10.2147/CCID.S449367. 38283798 PMC10821645

[64] Kimball AB, Jemec GBE, Sayed CJ, Kirby JS, Prens E, Ingram JR, et al. Efficacy and safety of bimekizumab in patients with moderate-to-severe hidradenitis suppurativa (BE HEARD I and BE HEARD II): two 48-week, randomised, double-blind, placebo-controlled, multicentre phase 3 trials. Lancet. 2024;403:2504–19. 10.1016/S0140-6736(24)00101-6. 38795716

[65] Martora F, Megna M. Bimekizumab for Hidradenitis Suppurativa from Trials to Real Life: A Review of the Published Literature. Clin Cosmet Investig Dermatol. 2025;18:3511–9. 10.2147/CCID.S565686. 41450452 PMC12730175

[66] Kirby JS, Okun MM, Alavi A, Bechara FG, Zouboulis CC, Brown K, et al. Efficacy and safety of the oral Janus kinase 1 inhibitor povorcitinib (INCB054707) in patients with hidradenitis suppurativa in a phase 2, randomized, double-blind, dose-ranging, placebo-controlled study. J Am Acad Dermatol. 2024;90:521–9. 10.1016/j.jaad.2023.10.034. 37871805

[67] Ackerman LS, Schlosser BJ, Zhan T, Prajapati VH, Fretzin S, Takahashi H, et al. Improvements in moderate-to-severe hidradenitis suppurativa with upadacitinib: Results from a phase 2, randomized, placebo-controlled study. J Am Acad Dermatol. 2025;92:1252–60. 10.1016/j.jaad.2024.12.046. 39909350

[68] McGaraughty S, Strasburg D, McCarthy R, Ciura S, Kouzehgarani GN, Wilper T, et al. Jak1 inhibition reduces acute allodynia induced by specific upstream cytokines in rats: implications for the onset of Jak1 pain modulation. Neurosci Lett. 2026;883:138667. 10.1016/j.neulet.2026.138667. 42336078

[69] Papp KA, Weinberg MA, Morris A, Reich K. IL17A/F nanobody sonelokimab in patients with plaque psoriasis: a multicentre, randomised, placebo-controlled, phase 2b study. Lancet. 2021;397:1564–75. 10.1016/S0140-6736(21)00440-2. 33894834

[70] Dogra S, Singh S. Assessing the clinical progress of the bispecific nanobody sonelokimab. Expert Opin Investig Drugs. 2025;34:253–8. 10.1080/13543784.2025.2500292. 40297934

[71] Qiu L, Hu F, Tao X. Research progress on the application of nanobodies in immunity and infectious skin diseases. Front Immunol. 2025;16:1705553. 10.3389/fimmu.2025.1705553. 41451206 PMC12728016

[72] Kim YJ, Sang J, Xiang Z, Shen Y, Shi Z, Nanobodies Y. Robust miniprotein binders in biomedicine. Adv Drug Deliv Rev. 2023;195:114726. 10.1016/j.addr.2023.114726. 36754285 PMC11725230

[73] Alavi A, Prens EP, Kimball AB, Frew JW, Krueger JG, Mukhopadhyay S, et al. Proof-of-concept study exploring the effect of spesolimab in patients with moderate-to-severe hidradenitis suppurativa: a randomized double-blind placebo-controlled clinical trial. Br J Dermatol. 2024;191:508–18. 10.1093/bjd/ljae144. 38576350

[74] Goldstein JD, Bassoy EY, Caruso A, Palomo J, Rodriguez E, Lemeille S, et al. IL-36 signaling in keratinocytes controls early IL-23 production in psoriasis-like dermatitis. Life Sci Alliance. 2020;3:e202000688. 10.26508/lsa.202000688. 32345660 PMC7190273

[75] Constantinou CA, Fragoulis GE, Nikiphorou E. Hidradenitis suppurativa: infection, autoimmunity, or both? Ther Adv Musculoskelet Dis. 2019;11:1759720X19895488. 10.1177/1759720X19895488. 31908656 PMC6937531

[76] Li Q, Liu S, Li L, Ji X, Wang M, Zhou J. Spinal IL-36γ/IL-36R participates in the maintenance of chronic inflammatory pain through astroglial JNK pathway. Glia. 2019;67:438–51. 10.1002/glia.23552. 30578562

[77] Kimball AB, Sawicki KT, Ackerman L, Lima H, Giamarellos-Bourboulis EJ, Zhan T, et al. Lutikizumab in Adults With Moderate to Severe Hidradenitis Suppurativa After Anti-TNF Therapy Failure. JAMA Dermatol. 2026;162:469–77. 10.1001/jamadermatol.2026.0155. 41848710 PMC13000743

[78] Ben Abdallah H, Bregnhøj A, Emmanuel T, Ghatnekar G, Johansen C, Iversen L. Efficacy and Safety of the Heat Shock Protein 90 Inhibitor RGRN-305 in Hidradenitis Suppurativa: A Parallel-Design Double-Blind Trial. JAMA Dermatol. 2024;160:63–70. 10.1001/jamadermatol.2023.4800. 38055242 PMC10701664

[79] Bregnhøj A, Thuesen KKH, Emmanuel T, Litman T, Grek CL, Ghatnekar GS, et al. HSP90 inhibitor RGRN-305 for oral treatment of plaque-type psoriasis: efficacy, safety and biomarker results in an open-label proof-of-concept study. Br J Dermatol. 2022;186:861–74. 10.1111/bjd.20880. 34748646

[80] Ambade A, Catalano D, Lim A, Mandrekar P. Inhibition of heat shock protein (molecular weight 90 kDa) attenuates proinflammatory cytokines and prevents lipopolysaccharide-induced liver injury in mice. Hepatology. 2012;55:1585–95. 10.1002/hep.24802. 22105779 PMC3342823

[81] Hu X, Li J, Fu M, Zhao X, Wang W. The JAK/STAT signaling pathway: from bench to clinic. Signal Transduct Target Ther. 2021;6:402. 10.1038/s41392-021-00791-1. 34824210 PMC8617206

[82] Shalaby M, Sahni R, Puebla D, Fernandez S. Modified PECS II Block for Axillary Hidradenitis Suppurativa. J Emerg Med. 2024;66:e701–3. 10.1016/j.jemermed.2024.01.007. 38762374

[83] Shahabuddin Y, Gleeson N, Maguire PJ. Surgeon-administered ilio-inguinal and pudendal nerve blocks for major vulval surgery: An observational study with visual analogue pain scoring. Eur J Obstet Gynecol Reprod Biol. 2022;268:87–91. 10.1016/j.ejogrb.2021.11.429. 34890844

[84] Urits I, Ostling PS, Novitch MB, Burns JC, Charipova K, Gress KL, et al. Truncal regional nerve blocks in clinical anesthesia practice. Best Pract Res Clin Anaesthesiol. 2019;33:559–71. 10.1016/j.bpa.2019.07.013. 31791571

[85] Eldabe S, Nevitt S, Hunter CW, Rosenow JM, Maden M, Goudman L, et al. Systematic review and network meta-analysis of randomized trial evidence of spinal cord stimulation for chronic pain. Reg Anesth Pain Med. 2026:rapm-2025-107068. 10.1136/rapm-2025-107068. 41494785

[86] O’Connell NE, Ferraro MC, Gibson W, Rice AS, Vase L, Coyle D, et al. Implanted spinal neuromodulation interventions for chronic pain in adults. Cochrane Database Syst Rev. 2021;2022. 10.1002/14651858.CD013756.pub2. PMC8638262 34854473

[87] Knotkova H, Hamani C, Sivanesan E, Le Beuffe MFE, Moon JY, Cohen SP, et al. Neuromodulation for chronic pain. Lancet. 2021;397:2111–24. 10.1016/S0140-6736(21)00794-7. 34062145

[88] Chapman KB, Sayed D, Lamer T, Hunter C, Weisbein J, Patel KV, et al. Best practices for dorsal root ganglion stimulation for chronic pain: guidelines from the American Society of Pain and Neuroscience. J Pain Res. 2023;16:839–79. 10.2147/JPR.S364370. 36942306 PMC10024474

[89] Gibson W, Wand BM, Meads C, Catley MJ, O’Connell NE. Transcutaneous electrical nerve stimulation (TENS) for chronic pain - an overview of Cochrane Reviews. Cochrane Database Syst Rev. 2019. 10.1002/14651858.CD011890.pub3. 30776855 PMC6379178

[90] Smith TJ, Wang EJ, Loprinzi CL. Cutaneous electroanalgesia for relief of chronic and neuropathic pain. N Engl J Med. 2023;389:158–64. 10.1056/NEJMra2110098. 37437145

[91] Johnson MI, Paley CA, Jones G, Mulvey MR, Wittkopf PG. Efficacy and safety of transcutaneous electrical nerve stimulation (TENS) for acute and chronic pain in adults: a systematic review and meta-analysis of 381 studies (the meta-TENS study). BMJ Open. 2022;12:e051073. 10.1136/bmjopen-2021-051073. 35144946 PMC8845179

[92] Ulschmid C, Serrano L, Wu R, Roth GM, Sokumbi O. African American race is a risk factor for severe hidradenitis suppurativa. Int J Dermatol. 2023;62:657–63. 10.1111/ijd.16428. 36183313

[93] Polaskey MT, Chovatiya R. Understanding disparities in hidradenitis suppurativa through social and structural determinants of health. JEADV Clinical Practice. 2024;3:945–61. 10.1002/jvc2.409.

[94] Barnes LA, Rinderknecht F-AB, Orenstein LAV, Naik HB. Global Pain Management Trends and Barriers to Care in Hidradenitis Suppurativa. Dermatology. 2026;242:252–63. 10.1159/000550402. 41758771 PMC13344115

[95] Hernandez Y, Gonzalez N, Ghanshani R, Castillo Valladares HB, Amerson EH, Naik HB, et al. Health System Experiences and Care Engagement in Latine Patients With Hidradenitis Suppurativa. JAMA Dermatol. 2026;162:151. 10.1001/jamadermatol.2025.5112. 41432947 PMC12728727

[96] Gupta AK, Economopoulos V, Mirmirani P, Magalhaes R. Understanding the evolving treatment landscape of hidradenitis suppurativa: an analysis of All of Us. PLoS One. 2025;20:e0331032. 10.1371/journal.pone.0331032. 40845034 PMC12373187

[97] Morden NE, Chyn D, Wood A, Meara E. Racial Inequality in Prescription Opioid Receipt — Role of Individual Health Systems. N Engl J Med. 2021;385:342–51. 10.1056/NEJMsa2034159. 34289277 PMC8402927

[98] Garg A, Papagermanos V, Midura M, Strunk A, Merson J. Opioid, alcohol, and cannabis misuse among patients with hidradenitis suppurativa: A population-based analysis in the United States. J Am Acad Dermatol. 2018;79:495–e5001. 10.1016/j.jaad.2018.02.053. 29499293

[99] Wang CX, Buss JL, Keller M, Anadkat MJ. Factors Associated With Dermatologic Follow-up vs Emergency Department Return in Patients With Hidradenitis Suppurativa After an Initial Emergency Department Visit. JAMA Dermatol. 2022;158:1378. 10.1001/jamadermatol.2022.4610. 36287553 PMC9607935

[100] Lipa K, Zając N, Witkowski G, Ciechanowicz P, Wiszniewski K, Szymańska E, et al. Hidradenitis suppurativa - biologic therapy and other available treatment options. Postepy Dermatol Alergol. 2023;40:518–28. 10.5114/ada.2021.112075. 37692279 PMC10485753

[101] Jin X, Gereau RW. Acute p38-mediated modulation of tetrodotoxin-resistant sodium channels in mouse sensory neurons by tumor necrosis factor-alpha. J Neurosci Off J Soc Neurosci. 2006;26:246–55. 10.1523/JNEUROSCI.3858-05.2006. PMC6674296 16399694

[102] Schäfers M, Lee DH, Brors D, Yaksh TL, Sorkin LS. Increased sensitivity of injured and adjacent uninjured rat primary sensory neurons to exogenous tumor necrosis factor-alpha after spinal nerve ligation. J Neurosci Off J Soc Neurosci. 2003;23:3028–38. 10.1523/JNEUROSCI.23-07-03028.2003. PMC6742101 12684490

[103] Leo M, Argalski S, Schäfers M, Hagenacker T. Modulation of voltage-gated sodium channels by activation of tumor necrosis factor receptor‐1 and receptor‐2 in small DRG neurons of rats. Mediators Inflamm. 2015;2015:124942. 10.1155/2015/124942. 26504355 PMC4609494

[104] Yao C-Y, Weng Z-L, Zhang J-C, Feng T, Lin Y, Yao S. Interleukin-17A Acts to Maintain Neuropathic Pain Through Activation of CaMKII/CREB Signaling in Spinal Neurons. Mol Neurobiol. 2016;53:3914–26. 10.1007/s12035-015-9322-z. 26166359

[105] Sun J-L, Dai W-J, Shen X-Y, Lü N, Zhang Y-Q. Interleukin-17 is involved in neuropathic pain and spinal synapse plasticity on mice. J Neuroimmunol. 2023;377:578068. 10.1016/j.jneuroim.2023.578068. 36948094

[106] Mills KHG. IL-17 and IL-17-producing cells in protection versus pathology. Nat Rev Immunol. 2023;23:38–54. 10.1038/s41577-022-00746-9. 35790881 PMC9255545

[107] Jiang X, Zhou R, Zhang Y, Zhu T, Li Q, Zhang W. Interleukin-17 as a potential therapeutic target for chronic pain. Front Immunol. 2022;13:999407. 10.3389/fimmu.2022.999407. 36248896 PMC9556763

[108] Kataria S, Patel U, Yabut K, Patel J, Patel R, Patel S, et al. Recent Advances in Management of Neuropathic, Nociceptive, and Chronic Pain: A Narrative Review with Focus on Nanomedicine, Gene Therapy, Stem Cell Therapy, and Newer Therapeutic Options. Curr Pain Headache Rep. 2024;28:321–33. 10.1007/s11916-024-01227-5. 38386244 PMC11126447

[109] Kaye AD, Armistead G, Amedio LS, Manthei ME, Ahmadzadeh S, Bernhardt B, et al. Evolving Treatment Strategies for Neuropathic Pain: A Narrative Review. Med (Mex). 2025;61:1063. 10.3390/medicina61061063. PMC12195047 40572751

[110] Zouboulis CC, Tzellos T, Kyrgidis A, Jemec GBE, Bechara FG, Giamarellos-Bourboulis EJ, et al. Development and validation of the International Hidradenitis Suppurativa Severity Score System (IHS4), a novel dynamic scoring system to assess HS severity. Br J Dermatol. 2017;177:1401–9. 10.1111/bjd.15748. 28636793

[111] Martorell A, Ingram JR, Sayed CJ, Bechara FG, Porter ML, Tzellos T, et al. Defining moderate disease and progression in hidradenitis suppurativa: an expert framework to unlock the window of opportunity for prompt treatment. Am J Clin Dermatol. 2026;27:217–26. 10.1007/s40257-026-01011-8. 41803418 PMC13032953

[112] Nikolakis G, Alpsoy E, Anzengruber F, Augustin M, Bechara FG, Becherel P-A, et al. Delphi consensus: First-line use of biologics and small molecules in hidradenitis suppurativa. J Eur Acad Dermatol Venereol JEADV. 2026. 10.1111/jdv.70264. 41580305

[113] Kouris A, Platsidaki E, Christodoulou C, Efstathiou V, Dessinioti C, Tzanetakou V, et al. Quality of Life and Psychosocial Implications in Patients with Hidradenitis Suppurativa. Dermatology. 2016;232:687–91. 10.1159/000453355. 28052274

[114] Centene Corporation. Clinical Policy: Adalimumab (Humira), Adalimumab-atto (Amjevita) 2016.

[115] Arora N, Skaggs E, Bicknell L. Limitations in medicaid coverage of biologics for the treatment of hidradenitis suppurativa. Arch Dermatol Res. 2024;316:619. 10.1007/s00403-024-03371-9. 39276219

[116] Louisiana Medicaid. Louisiana Uniform Prescription Drug Prior Authorization Form: Adalimumab-bwwd (Hadlima) 2023.

[117] Leoni MLG, Mercieri M, Viswanath O, Cascella M, Rekatsina M, Pasqualucci A, Caruso A, Varrassi G. Neuropathic Pain: A Comprehensive Bibliometric Analysis of Research Trends, Contributions, and Future Directions. Curr Pain Headache Rep. 2025;29(1):73. 10.1007/s11916-025-01384-1. PMID: 40183995; PMCID: PMC11971142. 40183995 PMC11971142

[118] Upshaw WC, Richey JM, Ravi G, Chen A, Ahmadzadeh S, Shekoohi S, et al. An overview of the safety and efficacy of LX-9211 in treating neuropathic pain conditions. Expert Opin Investig Drugs. 2024;33(8):829–37. 10.1080/13543784.2024.2376570. 38973395

[119] Upshaw WC, Soileau LG, Storey NR, Perkinson KA, Luther PM, Spillers NJ, Robinson CL, Miller BC, Ahmadzadeh S, Viswanath O, Shekoohi S, Kaye AD. An extract of phase II and III trials on recent developments in managing neuropathic pain syndromes: diabetic peripheral neuropathy, trigeminal neuralgia, and postherpetic neuralgia. Expert Opin Emerg Drugs. 2024;29(2):103–12. Epub 2024 Feb 29. PMID: 38410863. 38410863 10.1080/14728214.2024.2323193

